# C-Terminal Substitution of MDM2 Interacting Peptides Modulates Binding Affinity by Distinctive Mechanisms

**DOI:** 10.1371/journal.pone.0024122

**Published:** 2011-08-31

**Authors:** Christopher J. Brown, Shubhra G. Dastidar, Soo T. Quah, Annie Lim, Brian Chia, Chandra S. Verma

**Affiliations:** 1 Bioinformatics Institute, A*STAR (Agency for Science, Technology and Research), Biopolis, Singapore; 2 p53 Laboratory, A*STAR (Agency for Science, Technology and Research), Biopolis, Singapore; 3 Experimental Therapeutics Centre, A*STAR (Agency for Science, Technology and Research), Biopolis, Singapore; 4 Department of Biological Sciences, National University of Singapore, Singapore, Singapore; 5 School of Biological Sciences, Nanyang Technological University, Singapore, Singapore; Koç University, Turkey

## Abstract

The complex between the proteins MDM2 and p53 is a promising drug target for cancer therapy. The residues 19–26 of p53 have been biochemically and structurally demonstrated to be a most critical region to maintain the association of MDM2 and p53. Variation of the amino acid sequence in this range obviously alters the binding affinity. Surprisingly, suitable substitutions contiguous to this region of the p53 peptides can yield tightly binding peptides. The peptide variants may differ by a single residue that vary little in their structural conformations and yet are characterized by large differences in their binding affinities. In this study a systematic analysis into the role of single C-terminal mutations of a 12 residue fragment of the p53 transactivation domain (TD) and an equivalent phage optimized peptide (12/1) were undertaken to elucidate their mechanistic and thermodynamic differences in interacting with the N-terminal of MDM2. The experimental results together with atomistically detailed dynamics simulations provide insight into the principles that govern peptide design protocols with regard to protein-protein interactions and peptidomimetic design.

## Introduction

The tumour suppressor protein p53 is a transcription factor that plays an essential role in guarding the cell in response to a variety of stress signals through induction of cell cycle arrest, apoptosis or senescence [1], [2]. The activity of p53 is regulated by the E3-ubiquitin ligase MDM2. MDM2 inhibits p53 by preventing its interaction with the general transcription machinery and targeting it for ubiquitin-mediated degradation. MDM2 interacts with p53 through at least two regions: the N-terminus of p53 interacts with the N-terminal domain of MDM2 and the DNA-binding domain of p53 interacts with the acidic domain of MDM2 [3], [4]. MDM2 is overexpressed in many cancers, and is thought to be one of the primary causes of the inactivation of the p53 network in p53 wild type tumours [1]. In such cases, disruption of the MDM2∶p53 interaction has been shown to stabilize and activate the transcriptional activity of p53 leading to cell death or to G1/G2 cell cycle arrest [5], [6].

The molecular interaction of the binding of the MDM2 N-terminal domain to the p53 N-terminus (TA domain) is well understood. Several high-resolution crystal and NMR structures in complex with a variety of peptides and small molecules have been elucidated [7], [8]. These structural studies indicate that the residues F19, W23 and L26 of the TA domain of p53 are critical for binding to MDM2. Indeed most peptides, peptidomimetics and small molecules that have been developed have indeed emerged as a result of mimicking these interactions. However, it has been known for some time that the other residues in this sequence, such as the C-terminal residues, also modulate affinity [9]. Zondlo *et al*. hypothesized that this may originate in the ability of these regions to adopt a helical motif [10]. They found that the P27S substitution in a peptide corresponding to residues 12–30 of the WT p53 sequence gave rise to ∼200 fold improvement over the wild type sequence in the K_d_ (dissociation constant) of binding to the N-terminal domain of MDM2. CD and NMR suggested that the P27S substitution appeared to increase the helical propensity of the peptide. Computer simulations further complemented these findings and additionally demonstrated that this interaction can originate in multiple and distinct conformations of the peptide which include both partly extended and helical motifs at the C-terminal end [11].

More recently, several phage derived peptides have been derived and characterized with K_d_s that are of the same order of magnitude as that reported by Zondlo *et al.* [10] Of these peptides, two have either a T or S substitution in the P27 position, and further structural characterization revealed that their C-termini are in an extended α-helical conformation when bound to MDM2 [12], [13] in contrast to the extended form of the wild type p53 peptide complexed with MDM2 [14]. These high affinity peptides differ in their overall amino acid composition and so it is hard to distill out the contributions from individual amino acids. In contrast, we now show, through a combination of biophysical and molecular dynamics simulation methods that the binding affinities of two different MDM2 binding peptides can be further modulated by several orders of magnitude by just single amino acid substitutions. These changes lie at the C-terminus of the peptide and what is surprising is the observation that these peptides interact with MDM2 via two distinctive thermodynamic mechanisms. The observations made in this study should aid in the design of high affinity peptides. Coupled with emerging techniques in delivering peptides into cells, e.g. cell penetrating peptides or stapling, our observations should further aid the development of peptides into clinical leads.

## Materials and Methods

### Peptides synthesis

All Fmoc-protected amino acids, Hydroxybenzotriazole (HOBt), O-Benzotriazole-N,N,N′,N′-tetramethyl-uronium-hexafluoro-phosphate (HBTU) and Rink amide resin were purchased from Novabiochem (Germany). Trifluoroacetic acid (TFA) and anhydrous ethyl ether was purchased from Sigma Aldrich (USA). Dichloromethane (DCM), Diisopropylethylamine (DIPEA), *N*,*N*-Dimethylformamide (DMF), piperidine and HPLC grade acetonitrile were obtained from Merck (Germany).

Peptide amides were synthesized using a CEM Liberty (USA) automated microwave peptide synthesizer on Rink amide resin. All the peptides had their C-termini amidated. Fmoc deprotection was performed using 20% piperidine in DMF (v/v) at 75°C for 5 minutes at 40 W. Amino acid coupling reactions were performed in 5-fold molar excess of Fmoc-protected amino acids dissolved in DMF with activating reagents HBTU∶HOBt∶DIPEA∶amino acid (0.9∶1∶2∶1 equivalents). Coupling reactions were conducted over 10 minutes at 40 W and 75°C. Cleavage was performed using 5 ml of cleavage solution TFA∶water (95∶5 v/v) for 30 min at 40°C. Filtration was carried out and the resin was washed thrice with DCM to obtain the filtrate. The filtrate was concentrated under low-pressure centrifugal evaporation and the crude peptides were precipitated using ice-cold anhydrous diethyl ether. HPLC purification of peptide was performed using a Waters X-bridge C18 column (19 mm diameter, 150 mm length) at 215 nm wavelength. Separation was achieved by gradient elution of 25 to 45% solvent B (solvent A = 0.1% TFA in water; solvent B = 0.1% TFA in acetonitrile) at a flow rate of 5 ml/min. Molecular mass analysis was performed using Applied Biosystems Mariner Mass Spectrometer (USA) with electrospray ionization.

### Expression and purification of MDM2

DNA encoding residues Q18 to N125 of HDM2 were cloned as a NdeI/BamHI fragment into pET19b (Novagen). Recombinant clones carrying the correct insert were identified by DNA sequencing. Plasmid from the identified clone was introduced into E. coli BL21(DE3) and protein production in LB medium was induced with IPTG (1 mM) when the cell density reached OD600 between 0.4 and 0.6. Induced cells were grown at room temperature for 5 h, harvested, and then resuspended in buffer A (50 mM Tris pH 8.0, 10% sucrose). After cell disruption and centrifugation, supernatant was loaded onto a Ni-nitrilotriacetic acid (NTA) column and washed with 5 column volumes of 0.3 M NaCl, 50 mM Tris-HCl pH 8.0, 1 mM DTT. Hexahistidine tagged MDM2 (18–125) was eluted with a 1 M imidazole linear gradient. The protein was further purified by cation-exchange chromatography (Pharmacia Mono S 5/5, 1 ml/min, 50 mM Tris-HCl pH 7.0, 1 mM DTT) with a 1 M NaCl gradient. Protein concentration was determined using A280 with an extinction coefficient of 10430 M^−1^ cm^−1^.

### Isothermal Titration Calorimetry (ITC)

ITC experiments were conducted in a VP-ITC Unit (MicroCal, Northampton, MA). In a typical ITC experiment, 10 or 20 µM of MDM2 was loaded in the cell with 100 or 200 µM of peptide in the titrating syringe, depending on the binding affinities of compounds. All titrations were carried out in 1% DMSO. MDM2 was dialysed into Phosphate Buffered Saline (2.7 mM KCL and 137 mM NaCL, pH 7.4) with 0.05% TWEEN20 using SLIDE-A-LYZER (Pierce) cassettes with a MWCO of 3000. All peptides used were weighed out as solids and dissolved in DMSO. The stock peptide solutions were diluted to their working concentrations in the buffer used for dialysis of the protein. DMSO was carefully added to the MDM2 solution in the VP-ITC cell to buffer match it to the peptide solution. The titration experiments were performed at 20°C with an initial 2 µl injection with duration of 4 s, followed by 28 10 µl injections with a duration of 7.1 s. The spacing between each injection was 150 s. The stirring speed during the titration was 290 rpm. Data was analyzed using Microcal Origin software by fitting to a single-site binding model. Correction for the enthalpy of ligand dilution was carried out by subtracting a linear fit from the last three data points of the titration, after the interaction had reached saturation.

### Circular Dichroism (CD)

CD was measured on a JASCO J-810 spectropolarimeter and spectra were recorded in a 1 cm quartz cuvette (Helmer) in 5 mM sodium phosphate buffer (pH 7.0). Far UV CD spectra were recorded from 260 to 195 nm at a peptide concentration of 12.5 µM. The CD signal was converted to Delta Epsilons (Δε). CD spectra were recorded at a data pitch of 0.2 nm at 50 nm/min, a response time of 2 s and the bandwidth set at 2 nM.

### Computer simulations

The crystal structure (PDB code 1YCR) of the complex between MDM2 (residues 25–109) and a p53 peptide (residues 17–29) was used as a template to model the complexes studied [14]. The sequence of the 12/1 peptide was M_1_PRFMDYWEGLN_12_ and the equivalent sequence in p53 is Q_16_ETFSDLWKLLP_27_. The crystal structure (1YCR) of the complex between p53 and MDM2 does not contain the coordinates of the residue Q16. These were modeled using CHARMM22 parameters [15]. The residue E28 and N29 were not included. The final complex between the MDM2 and the 12 residue p53 peptide is referred to as p53WT hereafter. This was also used to construct the complexes between MDM2 and mutant p53 peptides resulting in P27S (p53Ser), P27A (p53Ala), P27T (p53Thr) and P27N (p53Asn). The same protocol was used to construct the MDM2-12/1 complex and its variants, wild type (12/1), N12S (12/1Ser), N12A (12/1Ala), N12T (12/1Thr), N12P (12/1Pro); the amino acid substitution of A, T and S were made to the C-terminal ‘Asn’ which is equivalent to the terminal Pro in the p53WT sequence. Patches were applied to both ends of the peptides, with the N-terminus acetylated and the C-terminus amidated.

Each complex was solvated using a box of TIP3P water molecules [16], maintaining a minimum thickness of a water layer of 9 Å. The net charge of the systems was neutralized by adding counter-ions (Cl^−^). After a brief energy minimization, the systems were subject to molecular dynamics (MD) simulations that included an initial heating to 300 K, followed by equilibration under constant pressure and temperature conditions (NPT). Finally, MD was run at constant temperature and volume conditions (NVT) for 20 ns for each system, leading to a combined simulation time of ∼200 ns; the 20 ns timescale was chosen based on our earlier work on this complex which has successfully reproduced experimental data [17]. Particle Mesh Ewald (PME) [17] was used to calculate long range electrostatics and a 12 Å cutoff was applied for short ranged non-bonded interactions. SHAKE [18] was applied to freeze the vibrations of bonds involving hydrogen atoms. The figures (molecular structures) were prepared using Pymol [19] while the movies were prepared using VMD [20].

## Results

### Single amino acid substitutions dramatically increase the affinity of the MDM2 binding peptides

**Figure 1:**
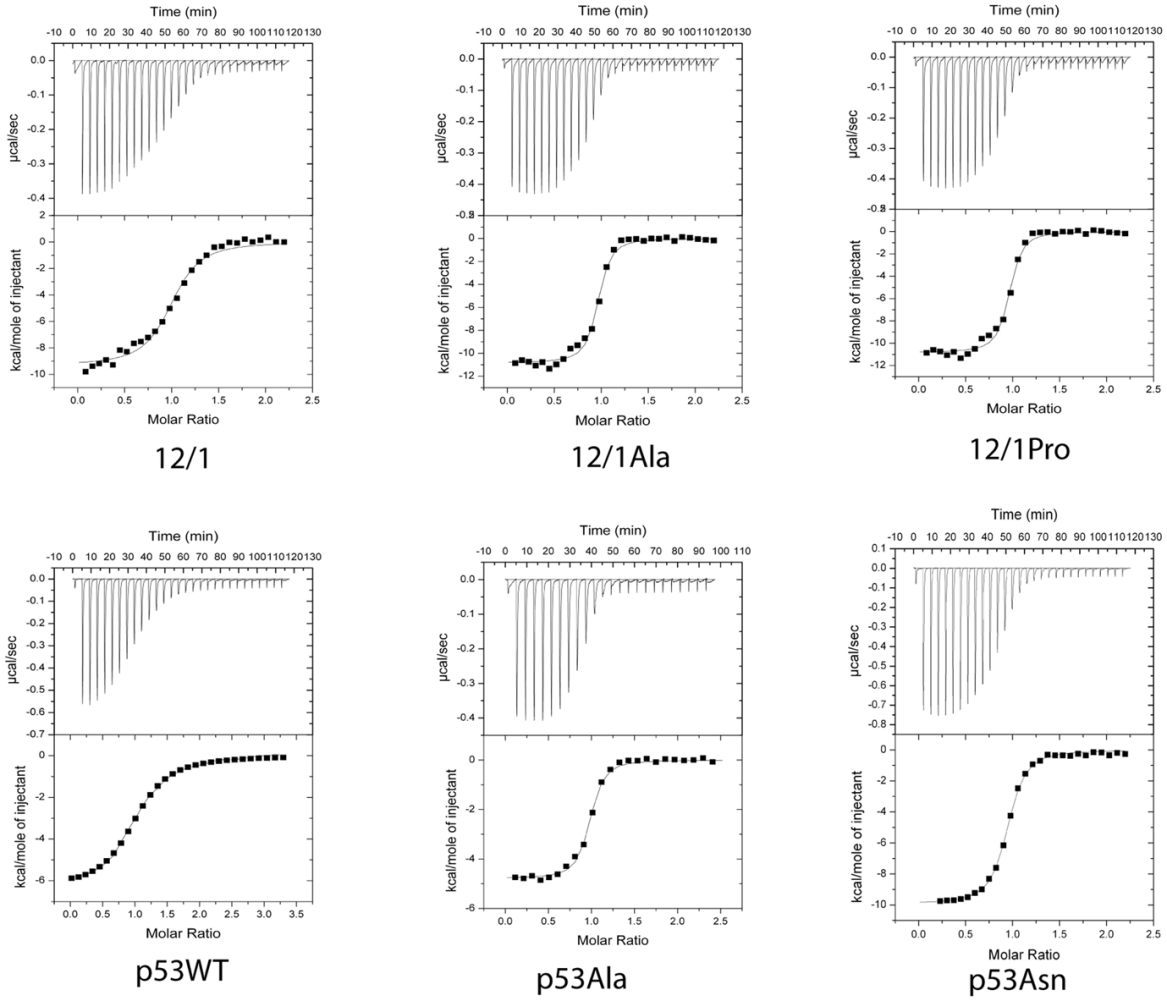
Binding Isotherms of MDM2 with the MDM2 interacting peptides. Isothermal Calorimetry titrations of selected MDM2 binding peptides against MDM2.

The p53WT peptide, as determined from ITC studies (see Figure 1 and Table 1), has a K_d_ of 1543.21±89.97 nM. The p53 derivative set of peptides displayed substantially stronger binding to hexahistidine tagged MDM2 (18–125) compared to the original peptide, with K_d_s ranging from ∼13-fold to 36 fold lower than for the wild type sequence. The p53Thr peptide was the most potent peptide, with a K_d_ of 38.76±7.43 nM. All three derivative peptides (see Table 1) had K_d_s significantly lower than that of racemic Nutlin (K_d_ was determined to be 201.61±60.85 nM). The 12/1 peptide binds to MDM2 with a K_d_ of 239.81±53.79 nM which is ∼6.5 fold lower than that of the p53WT peptide and is similar to the K_d_ of racemic Nutlin (Table 1). Again all three amino acid changes increased the potency of the peptide with 12/1Ser and 12/1Thr having the lowest apparent K_d_s of 18.83±5.03 nM and 22.88±4.21 nM respectively. Compared to the equivalent substitutions in the p53 derived peptides, the 12/1 derived peptides do not improve binding as dramatically when compared to their parent peptide. However when the equivalent changes are compared to each other, the 12/1 derived peptides have lower apparent K_d_ values than their p53 counterparts. The 12/1Ser peptide has a K_d_ value that is ∼4 fold lower that the p53Ser while the other two other peptides in the set are ∼ two-fold lower in their K_d_ than their equivalent p53-derived peptides.

**Table 1:** Calculated K_d_s and thermodynamic parameters for the interactions between MDM2 and the derivative peptides.

Ligands	ΔG(kCal/mol)	ΔH(kCal/mol)	TΔS(kCal/mol)	K_d_ (nM)
p53WT	−7.79±0.03	−6.34±0.07	1.45	1543.21±89.97
p53 Ser	−9.54±0.08	−5.08±0.04	4.45	75.76±10.45
p53Thr	−9.93±0.10	−6.33±0.05	3.57	38.76±7.43
p53 Ala	−9.31±0.09	−4.79±0.05	4.54	112.11±17.93
P53 Asn	−9.076±27	−9.71±0.04	−0.62	168.07±9.24
12/1	−8.87±0.12	−9.32±0.19	−0.44	239.81±53.79
12/1Ser	−10.35±0.14	−7.84±0.13	2.52	18.83±5.03
12/1Thr	−10.24±0.10	−8.22±0.06	2.03	22.88±4.21
12/1Ala	−9.82±0.12	−10.83±0.13	−1.00	47.17±11.07
12/1Pro	−8.94±0.09	−10.35±0.13	−1.40	213.22±36.59
Nutlin Racemic	−8.97±0.15	−6.10±0.16	2.88	201.61±60.85

ITC determined values for enthalpy of binding, entropy of binding, peptide dissociation constant and the binding free energy. The binding fee energy was calculated from the K_d_ using ΔG = −RTlnK_a._ Experiments were carried out at 293 K.

A further set of peptides were designed to see if the Asn residue located at the C-terminus of the 12/1 peptide would lead to an improvement in the binding of the p53WT peptide if it was used to replace the Pro residue. The resultant p53Asn residue bound MDM2 with an apparent K_d_ of ∼168.07±9.24 nM which is very similar to the apparent K_d_ of the 12/1 peptide of 239.81±53.79 nM. However if the Asn C-terminal residue of the 12/1 peptide is changed to Pro there is no significant change in the K_d_ of the new peptide compared to the parent peptide. This perplexing result indicates a subtle difference in the modes of interaction with MDM2 between the 12/1 and p53 based families of peptides.

The two groups of peptides vary in the thermodynamics of their interactions with MDM2. The p53 family members have a ΔH_binding_ that ranges from −4.8 kcal/mol to −9.7 kcal/mol (Table 1), whilst for the 12/1 peptide group it ranges between −7.8 kcal/mol and −10.8 kcal/mol (Table 1). The interaction of the members of the 12/1 family of peptides is primarily enthalpically driven compared to the p53 group, which have a more favorable entropic component. Interestingly the p53Asn peptide departs from this observation as it has the largest binding enthalpy of the p53 based peptides and an unfavourable entropic component. Also 12/1Pro has the most unfavourable entropic component out of the 12/1 peptides. The Ser, Thr and Ala modifications in the p53 family display a substantial increase in affinity compared to the parent p53WT because of entropic stabilizations. A similar mechanism underlies the Ser and Thr mutants of the 12/1 peptides. However the Ala modification in 12/1 leads to a big increase in the enthalpy and hence affinity for MDM2.

### CD studies reveal increased helicity in p53 derivative peptides compared to the 12/1 derivative peptides

**Figure 2:**
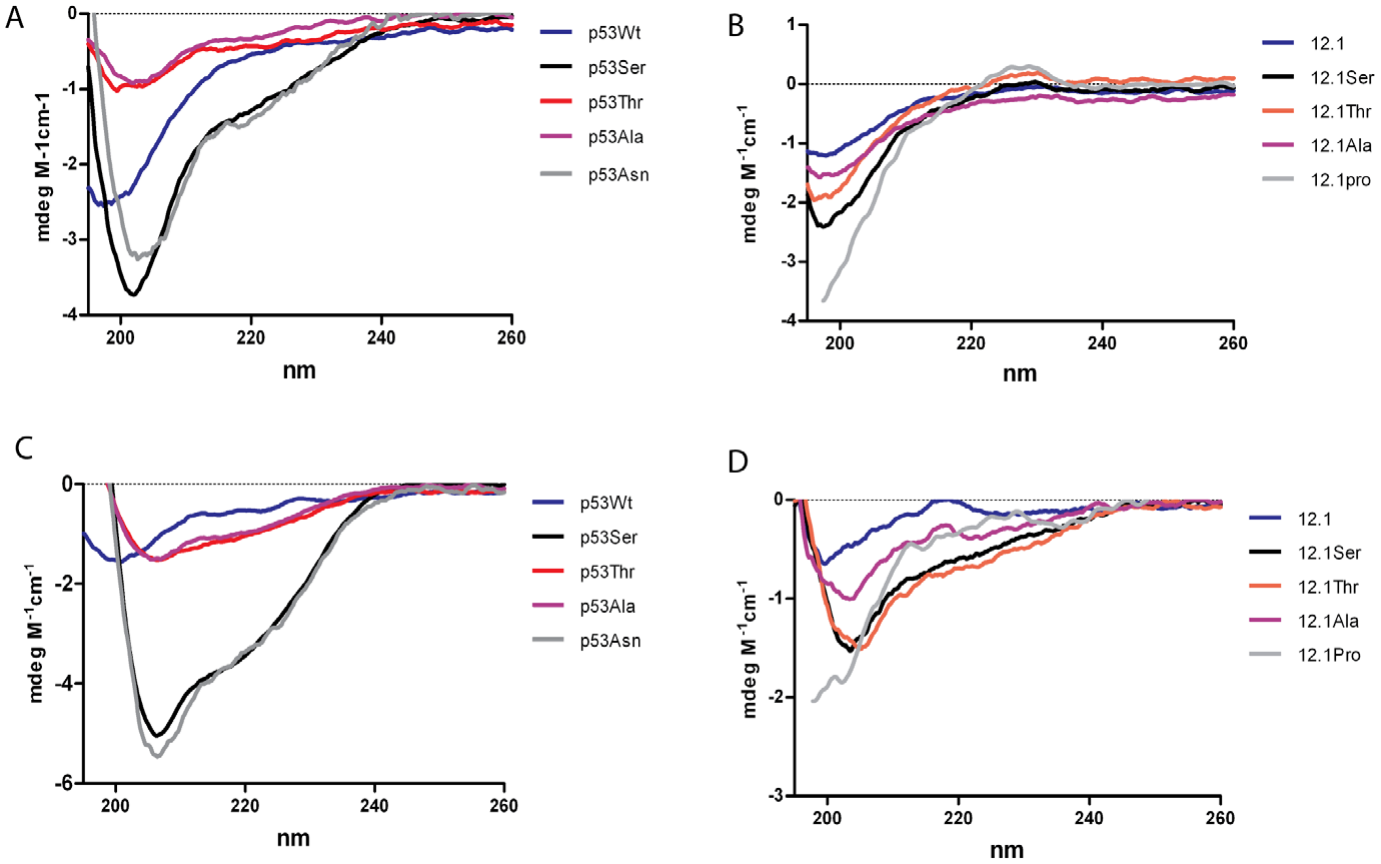
CD spectra of MDM2 interacting peptides free in solution. (A) CD spectra of the peptides P53Ser, p53Thr, p53Ala, p53ASN and p53WT in 5 mM Sodium Phosphate buffer pH 7.0 (C) and with the addition of 30% TFE. (B) CD spectra of peptides 12/1Ser, 12/1Thr, 12/1Ala, 12/1Asn and 12/1 in in 5 mM Sodium Phosphate buffer pH 7.0 and (D) with the addition of 30% TFE.

To investigate the molecular basis for the observed increase in affinity of the p53 and 12/1 derivative peptides we first focused on their conformational states in solution for which we carried out CD spectroscopic studies. The CD spectra of p53WT when compared to the CD spectra of p53Ser (Figure 2A) shows that the minima of the CD signal for the latter had undergone a small red shift (202 nm) in comparison to the p53WT (197 nm); it also exhibited a significant increase in the CD signal at 222 nm. These spectra indicate that the p53Ser peptide is substantially more α-helical than the p53WT peptide. Figure 2A also shows the CD spectra of p53Thr and p53Ala when compared to the p53WT peptide do not undergo a significant increase in the CD signal at 222 nm. However the spectra of the two daughter peptides do show a small red shift (201 nm and 202 nm respectively) in the minima and a decrease in the intensity of the CD signal observed at the minima as well, suggestive of increased helicity when compared to p53WT.

When the p53WT peptide and its derivatives are examined under the same buffer conditions but with the addition of 30% TFE, the CD spectra of the p53 derivative peptides show significant helix formation with an increase in the intensity of the CD signal at 206 nm and 222 nm and a sharp maxima at 195 nm (see Figure 2). Again the p53Ser peptide exhibits the strongest helical signature, with the CD signal intensity reaching −5 and −4 at 208 nm and 222 nm respectively (see Figure 2). This data confirms the observations by Zondlo et al [10] that the Pro27Ser mutation does indeed increase helix formation in the peptide. The CD signals of the other peptides are less intense. The CD spectra of the p53WT in 30% TFE shows the features of the p53Ala and p53Asn peptides in 5 mM Sodium Phosphate buffer, with a small red shift of the CD spectra minima and a decrease in its intensity (Figure 2). In general, the CD spectra suggest that the p53-derivative peptides have a higher propensity to form an α-helix than p53WT.

The CD spectra of the 12/1 family of peptides in Sodium phosphate buffer pH 7.0 are very similar to that of the p53WT peptide, and are typical of peptides in a coil formation exhibiting a minima at 197 nm (see Figure 2). However, when the same CD spectra are recorded with the addition of 30% TFE, all four spectra undergo distinct changes. The 12/1 peptide undergoes a slight red shift of the spectra minima (200 nm) and a decrease in the signal intensity of the minima which is similar to that seen with the p53WT peptide in 30%TFE. The 12/1 Ala peptide undergoes a larger red shift (203 nm) whilst the 12/1Thr and 12/1Ser peptides exhibit the red shift (205 nm and 204 nm respectively) as well as an increase in the CD intensity at 222 nm (see Figure 2). The 12/1Thr peptide has a slightly more intense CD signal at 222 nm and a greater red shift of its minima than the 12/1Ser, indicating greater helical formation. From the CD spectra of the 12/1 family of peptides, the 12/1Ser and 12/1Thr peptides display the greatest propensity for helix formation, followed by 12/1Ala and finally the parent peptide 12/1.

The CD data indicates that the amino acid substitutions within the p53WT sequence are clearly more α-helical inducing than within the phage optimized sequence 12/1, with larger red shifts being observed in buffer alone and in 30% TFE. Although both families are helical to differing extents, it is clear that the effect each substitution has is context dependent. These observations suggest that the increased affinities of the peptides over their parent peptides may originate in greater pre-structuring or pre-organization into helical motifs prior to binding MDM2. And yet, the fact that the 12/1 peptides are much less helical in nature than the p53 derivative peptides also suggests that the increased affinity of the 12/1 peptides compared to the p53 peptides will arise from additional factors.

The spectra of the two peptides, p53Asn and 12/1Pro, where the C-terminal residues have been exchanged, were also recorded. The p53Asn peptide produced spectra remarkably similar to that of p53Ser in both Sodium phosphate pH 7.0 buffer and with the addition of 30% TFE, although it did exhibit a slightly greater red shift in its minima in the absence of TFE. In contrast, the 12/1Pro, in both sets of conditions, possesses a random coil configuration and shows negligible helix formation.

### Computer simulations of the interactions between MDM2 and the derivative peptides

**Figure 3:**
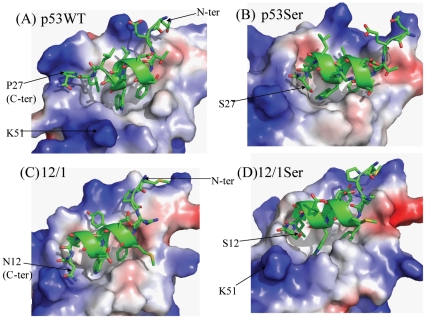
Representative snapshots depicting helix formation in the MDM2 interacting derivative peptides and their packing against the surface of MDM2. Representative snapshots to show that non-Proline substitution at the C-terminal end of the ligand promotes helix formation. Also shown is that the bulkier Asn side chain is accommodated differently compared to a smaller side chain like Ser when the peptide is in a helical conformation. The positively and negatively charged regions on the MDM2 surface have been shown with blue and red colors respectively.

**Figure 4:**
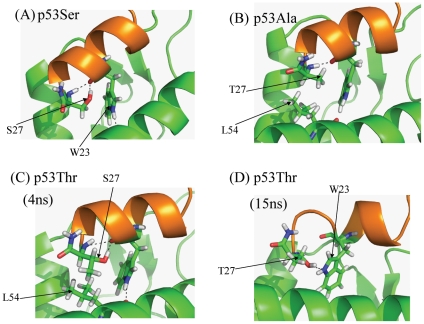
Representative snapshots from the computer simulations of the p53 family based peptides and their interactions with MDM2. Representative snapshots from the trajectories of MDM2 complexed with the p53 mutants: (A) P27S (B) P27A (C) P27T and (D) P27T. Two different snapshots have been shown of P27T to show significantly different interactions.

The residues 16–27 of wild type (WT) p53 peptide (Q_16_ETFSDLWKLLP_27_) are equivalent to residues 1–12 of the 12/1 peptide (M_1_PRFMDYWEGLN_12_). The template for modeling of all complexes was the crystal structure of MDM2-p53 (PDB code 1YCR; ([14])), where residues 19–25 of the p53 peptide form an α-helix, and the other residues are unstructured. In all the p53 mutant peptides modeled, the region 19–25 was assumed to be helical to begin with and rest of the peptide chain (residue 16–18 and 26–27) had no defined secondary structure. Similarly, for the 12/1 peptide and its mutants, residues 4–10 were modeled as α-helical, resembling closely the backbone of the p53 peptide in the crystal structure, while the remainder of the residues were unstructured, but shared a conformation similar to that of the p53 peptide complexed to MDM2 in the crystal structure. A general trend observed from the simulations was that the last two C-terminal residues of the peptides undergo a transition from an extended strand conformation to a helical conformation within a few nanoseconds (see Movies S1). This change in peptide conformation, coupled with the flipping of Y100 (MDM2) towards the ligand binding pocket, creates a more complementary fit for the peptide in the MDM2 binding pocket as has been reported previously [11], [13], [21], [22]. Without the rotation of the Y100 side chain, the C-terminal residues in the peptide would not make any specific contacts with MDM2. The simulations show that the formation of a helical conformation at the C-terminal is favoured and allows for better packing against the surface of MDM2, except in the presence of Pro (see Figure 3). The helical conformation is associated with intrahelical hydrogen bonds (see Figure 4) that the last two residues form and also appears to optimize the hydrophobic packing against the flipped Y100 side chain. This flipping of the Y100 side chain is additionally enthalpically favoured because it engages the hydroxyl of the Y100 in hydrogen bonds with the peptide backbone. Figure 3 shows that smaller side chains like Ser orient towards the MDM2 peptide binding cleft whereas larger groups like Asn point away from the binding cleft, towards K51. This area of MDM2 is polar in nature and stabilizes the preferred conformations of Asn and Ser. On the other hand the Ala side chain is unable to form interactions in this polar pocket of MDM2 in a manner that Ser/Thr can, and results in larger fluctuations in this region (see Movies S1). The N-terminal ends of the peptides are largely unstructured when bound to MDM2. For p53WT, the interaction of E17 with K94 of MDM2 prevents the formation of secondary structure in the region Q16-T18 of the peptide even though the helical propensities of these residues are high: Q16 (1.11), E2 (1.51), T (0.83), F(1.13). In a separate study, this region has been shown to undergo a conformational change to a helix, arising from long range electrostatic interactions [23] and is indicative of the plasticity of the underlying energy surface. In the case of 12/1, the E17 of p53WT is substituted with Pro which prevents any major conformational rearrangements. In addition, residue R3 of 12/1 interacts with a negatively charged region on the MDM2 surface (Figure 3) which increases the electrostatic component of the interaction between 12/1 and MDM2 thus making any conformational change energetically expensive.

### Two distinct thermodynamic systems of interactions between p53 and 12/1 based peptides with the MDM2 N-terminus

**Figure 5:**
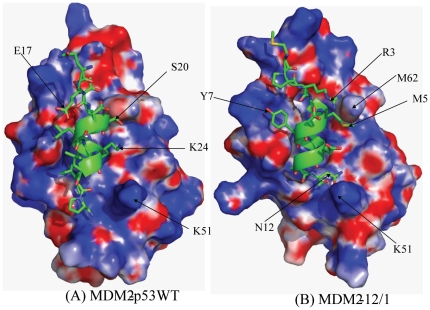
Snapshot showing packing of the p53WT and 12/1 against an electrostatic surface representation of the MDM2. A) Diagram shows the packing of p53WT peptide against an electrostatic surface representation of MDM2 (blue corresponds to positive and red to negative). B) Diagram shows that the 12/1 peptide packs against MDM2 with better electrostatic complementarity.

The major difference between the two groups of peptides is that the ΔH_binding_ for the p53 peptide family members (Table 1) ranges from −4.8 kcal/mol to −7.0 kcal/mol whereas for the 12/1 peptide group it ranges between −7.1 kcal/mol and −10.8 kcal/mol. The 12/1 group is enthalpically driven compared to the p53 group (see Table 1 for ITC data), although the conformations of the two groups in their complexed states are largely similar; this trend is also mirrored in the computations. The different enthalpies may originate, at least partly, in the observation that there appears to be greater electrostatic complementarity between the 12/1 peptides and MDM2 (see Figure 5) and also in the total electrostatic energies of the complexes which are about 15% stronger for the 12/1 peptide-MDM2 complex.

**Figure 6:**
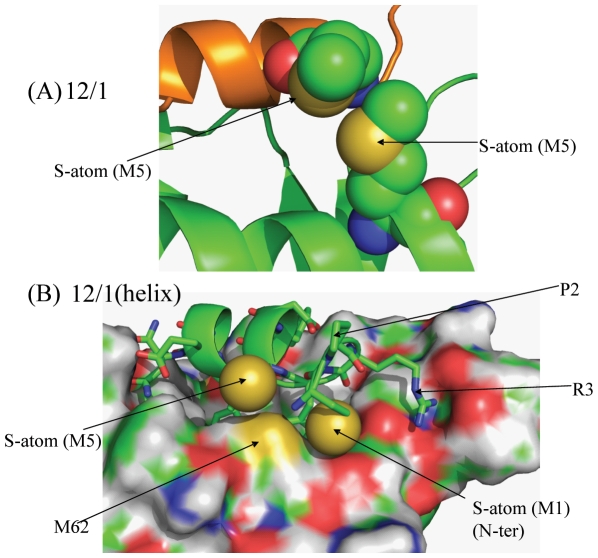
12/1 peptide has the potential to form extra packing interactions with MDM2. (A) The equivalent position of S20 of the p53 family has been replaced by M5 in the 12/1 peptide family. Residue M5 packs much more favorably against M62 of MDM2 than the S20 residue in the p53 based peptides. (B) If the first three residues of the 12/1 family are modeled as a helix, even though this was not observed in the simulation, the M1 residues would also pack favorably against the M5 12/1 residue of 12/1 and the M62 residue of MDM2.

In the 12/1 series, while residue M1 does not form any specific interactions with MDM2, residue M5 forms favourable van der Waals interactions with the M62 (MDM2) side chain (Figure 6). In p53, the equivalent position is occupied by S20 which cannot optimally pack with M62 and form such favourable hydrophobic interactions. In the 12/1 peptide, if this region is modeled as a helix (a representative snapshot was taken from the trajectory to model the residues M1-R3 as α-helical followed by some energy minimization) the interactions of R3 with the MDM2 are retained but a new cluster of van der Waals interactions are formed by the M1 and M5 residues of the peptide with M62 of MDM2 (Figure 6). The helical conformation described was not accessed during the 20 ns MD simulation and suggests that much longer timescales are needed for these transitions.

### Substitution of the C-terminal residue modulates the energy of binding of the MDM2 interacting peptides

**Figure 7:**
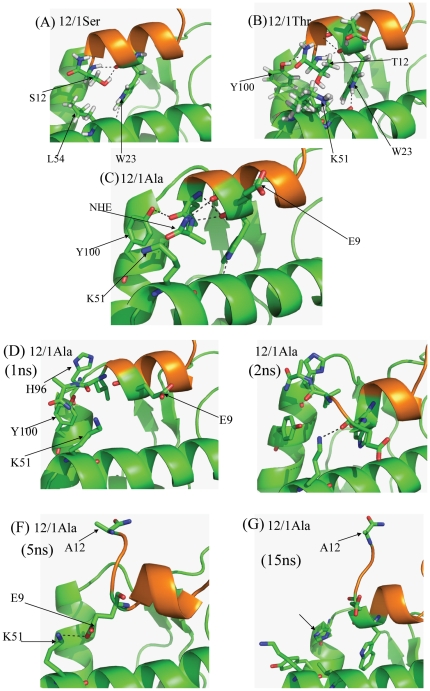
Representative snapshots from the trajectories of 12/1 mutant peptides complexed with MDM2. Snapshots from the trajectories of 12/1 mutant peptides complexed with MDM2. The (A) N12S, (B) N12T and (C) N12A MD simulations were started with a modeled helix at the C-terminus. Figures 7D) to 7G) show different orientations of the C-terminus of the N12A 12/1 mutant at different time points of the MD simulation trajectory (also see Movies S1).

For both groups of peptides, modulation of the free energy is determined by the type of substitution at the C-terminal residue. However the mechanism by which this modulation occurs in both groups shares several similarities. The Ser and Thr mutant peptides which are more helical in the CD data (Figure 2) have much higher entropic benefits when binding to MDM2. However, the 12/1Ala derivative does not undergo an entropically favourable interaction with MDM2. This may arise from increased fluctuations in the bound state. The higher helicity of the 12/1Ser and 12/1Thr peptides reduces the entropic cost upon transition from uncomplexed to MDM2-complexed states. Simulations show (see Figure 7) that the Ser side chain makes the peptide more prone to forming a helix by forming a hydrogen bond with the backbone of W23 (and also occasionally with the side chain NH of W23). The Thr substitution also appears to increase helicity via this mechanism (see Figure 7). This mechanism also applies to the Ser and Thr derivatives in the p53 family. Interestingly in the 12/1 family the Thr peptide has higher helical propensity, despite the presence of a methyl group which should interfere with the intrahelical hydrogen bonding. However the observed difference in helicity is probably due to the effect of the different peptide sequences and local packing effects.

The p53Ala and 12/1Ala derivative peptides behave differently to the Thr/Ser derivative peptides in the MD simulations with the C-terminal part of the Ala derivative peptides not undergoing a transition from the extended conformation to a helical confirmation during the simulation. In general Ala is helix promoting and has a very high helical propensity. However with Ala located at the C-terminus (N12A of 12/1 and P27A for p53) its effects on the local secondary structure of the peptide are reduced. It cannot form a hydrogen bond to W23/W8 (p53 and 12/1 peptides respectively) in the manner that Ser/Thr residues do. However if the C-terminal section of the Ala derivative peptide is modeled initially as α-helix then a 10 ns MD shows that it remains stable there. The helical conformation places the Ala residue in the vicinity of a highly polar environment on the surface of MDM2. The 12/1Ala has appears to stable in both situations, contributing −51.1 kcal/mol (extended) and −58 kcal/mol (helix) of the total stabilization energy [E_mm_(Total)] from MMGBSA calculations. The larger stability of the helical conformation arises from larger hydrophobic packing. Therefore the transition of the C-terminal region of the Ala derivative peptide from an extended to a helical conformation may be characterized by a larger energy barrier, and this may explain the lack of a spontaneous transition as observed for the other peptides.

According to the Chow Fasman scale, residues W23 to L26 in the p53 template peptide are all helix promoting. However, the presence of G10 (equivalent to L25 of p53) in the 12/1 peptide family drastically reduces the helical propensity and incorporates flexibility that in turn enables the C-terminus of 12/1Ala to remain in an extended conformation. It is this flexibility that allows the 12/1 family based peptides to pack more optimally against MDM2 and which is reflected overall by higher enthalpies of binding. This flexibility allows the C-terminal Ala to pack optimally against MDM2 (See Figure 7 and Movies S1). The extended conformation of the 12/1 derivative peptide encourages the formation of a salt bridge between E9-K51 and this is reflected in the much higher enthalpic gain upon complex formation (see Figure 7f). However the peptide simulations, with C-terminal region of 12/1Ala initially modeled as a helix, shows that the C-terminal region of the peptide remains stable as a helix throughout the simulation. The salt bridge between E9 and K51 is lost due to a larger spatial distance, but it seems that the structure tolerates the loss of the salt bridge, compensated for by the hydrophobic packing of the Ala residue.

The 12/1 peptide family also contains a Y (helix propensity 0.69) at position 7, which along with the glycine at position 10, causes the peptide to be more flexible and as a result enhances the entropic difference between the uncomplexed and complexed states of the peptide. This is in agreement with the observation that the 12/1 peptide group has a less favourable entropic component in their interactions with MDM2.

**Figure 8:**
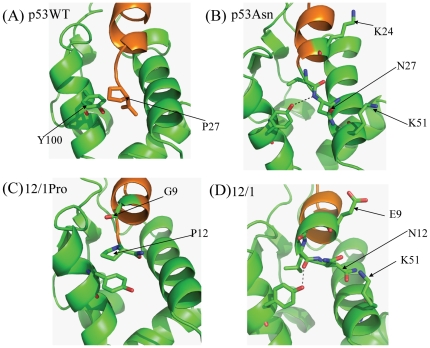
Representative snapshots from the trajectories of the WT p53 and the two C-terminal Pro substituted peptides illustrating the different interactions formed with MDM2. Representative snapshots from the trajectories of (A) WT p53 (B) P27N mutant of p53 (C) N12P derivative of the 12/1 family and (D) the 12/1 peptide complexed with MDM2 showing the different interactions formed by similar substituent amino acids at the C-terminal end of two peptide families.

For the proline derivative peptides the simulations show that they interact with MDM2 in an extended conformation at the C-terminus. However due to the higher flexibility of the 12/1Pro peptide the P12 residue is able to form stacking interactions with the Y100 ring (see Figure 8) that are not observed in the p53WT peptide. In the p53WT peptide there is still a tendency to form this interaction but due to the higher rigidity imposed by the helix favoring L25 and L26 residues the P27 and Y100 ring do not optimally pack against each other. For this reason the N12P mutation in 12/1Pro causes a decrease in the ΔH that seems to more than compensate for the loss of packing in the helical 12/1 peptide (which has N at position 12) and the change of orientation in Y100. However the Pro to Asn change in the p53 peptide family causes the peptide to pack more tightly and efficiently against MDM2, which leads to the slight enthalpic gain.

## Discussion

The N-terminus of MDM2 exhibits conformational plasticity that allows it to accommodate peptides in several different orientations. Recent papers have reported the discovery and development of peptides with nanomolar affinities that interact with MDM2 and also bind tightly to MDM4. Our studies have focused on the mechanism by which these peptides interact with MDM2 and how these peptides can be converted from the low micromolar and high nanomolar binding affinities to much more potent binding entities. Interestingly this conversion can be engineered by a simple mutation of the C-terminal residue in a 12 mer peptide to either a Thr, Ser or Ala. However intriguingly a destabilizing Pro at the C-terminus of the p53 peptide appears to lose the destabilization in the 12/1 peptide sequence, thus highlighting the importance of the context in which the mutation is made. This, together with differences in the helical propensities of a series of C-terminal mutants of the two peptides has demonstrated the existence of two distinct modes of interactions. One mode is much more enthalpically driven and correlates with peptides that have a lower helical propensity (the 12/1 family) and the other mode is more entropically driven and correlates with peptides that are more helical in nature (the p53 family). This makes sense since the p53 family has more preformed helical motifs in solution that reduces the entropic penalty upon sequestration by MDM2 while the 12/1 peptides are more flexible and hence “morph” better into the surface of MDM2, enhancing the enthalpic gains [24]. Molecular dynamics simulations suggest that the high affinity binders of both peptide families gravitate towards forming a stable C-terminal helix when bound to MDM2. These conformations are characterized by helical motifs that are associated with high affinity and the Y100-in state (Y100 points in towards the binding cavity). In contrast, peptides that prefer to form an extended C-terminal strand are characterized by the Y100-out state and have a lower affinity. These observations highlight that two distinct thermodynamic modes of interactions can be engineered to create more potent peptides via similar substitutions. However exceptions such as the effect of the inhibitory Pro in the p53 Wt sequence compared to its lack of effect on the 12/1 peptide highlight the complex and subtle interplay that is associated with the mutual modulation of the peptides and the protein surface.

The general consensus is that preorganizing a ligand into its bound conformation will reduce the entropic penalty upon binding, and is borne out generally in our system. But the picture may be far more complex than the simplistic two-state paradigm. First of all, a question arises as to the nature of helicity and its assumption in this process. CD spectroscopy tells us that these peptides assume helical conformations in solution. But what is not known is how different are these helices between the free and bound states and how much the protein and peptides modulate each other on binding [21], [25]. Our own thermodynamic data is chacaterized by several puzzling features. Why is it that in the p53 family peptides, the Ser, Thr, Asn and Ala mutants display helicity in solution, but in contrast the binding of Asn is entropically unfavourable while that of the other two very favourable. The answer lies partly in the fact that entropic changes involve many factors that simulations and even experiments are unable to account for. These factors can include the displacement of interfacial/buried water molecules (estimated to contribute up to 2 kcal/mol/water) [26], [27], burial of a water molecule [28] (estimated to contribute up to 1 kcal/mol) or increase in the conformational entropy of the complex [29]. The resolution of the current simulations did not enable us to succeed in computing differences that could provide a statistically robust mechanism behind the observed changes in entropy. Indeed, parameters such as fluctuation differences for example did not reveal any significant correlations, nor did analysis of hydration. In a detailed study combining NMR and simulations [30] the authors conclude that binding energetic differences between peptides to the same receptor can result from very small, subtle changes including hydration effects, that are really not readily quantifiable by simulations or experiment; some progress is being made [27]. Nevertheless, visual examination of the simulations provide some explanation. Upon complexation, the Asn mutant adopts a conformation that is extended at the C-terminus compared to that adopted by Ser/Thr. This clearly suggests that the Asn mutant goes from an unbound helical state to an extended state when bound and could account for the opposite trend in entropy even as it makes more enthalpic gains in the extended state. Of course it is not clear what the extent of helicity is in the bound state, given the resolution of CD spectroscopy and whether a small extension at the C-terminus in the unbound state will be detectable or not. Also it is likely that there are subtle changes in hydration at the C-terminus between the Asn and the Ser/Thr systems. In the case of Ser, Thr and Ala, the peptide is helical in the bound state and thus may result in the release of waters that would be embedded in the extended state, which is the case of Asn. However, the wild type is extended too and yet undergoes a large entropic gain. Close inspection reveals that while the Pro in the wild type enables the extended conformation of the C-terminus of p53 to stick to the side of MDM2, thus leading to an increase in the entropy of the bound state [11], the extended C-terminus in Asn does not stick to the surface but instead “hovers embedded in the cleft and interacts with the N-terminus of MDM2. This leads to the higher enthalpy and at the same time compacts the structure of the complex possibly leading to a decrease in the entropy. Indeed, local packing effects can have subtle origins and yet dramatic effects as was evidenced in a study where the mere change of an amino acid in a peptide from Thr to Ser renders the FHA domain incapable of recognizing the peptide [31]. In conclusion, the current work further dissects and highlights the subtle nature of protein-peptide interactions through a study of the interactions between the N-terminal domains of MDM2 and “designed” p53 peptides, and we hope our findings will further stimulate enquiry and the design of peptidomimetic molecules with more “rational” insights.

## Supporting Information

Movies S1 Movies of MD simulation trajectories.
(DOCX)
