# Supplementary material for: C-Terminal Substitution of MDM2 Interacting Peptides Modulates Binding Affinity by Distinctive Mechanisms
Source: PLoS One. 2011 Aug 31;6(8):e24122. doi: 10.1371/journal.pone.0024122 (PMC3164098; doi:10.1371/journal.pone.0024122)
Supplement: Movies S1 — Movies of MD simulation trajectories. (DOCX) [file pone.0024122.s001.docx]

**Supporting Information:** Movies of MD simulation trajectories are available from <http://web.bii.a-star.edu.sg/bmad/peptide-2010/>. Movies are in MPG format.
